# Bilateral brachial plexus block in a patient with cervical spinal cord injury: A case report

**DOI:** 10.1097/MD.0000000000021126

**Published:** 2020-07-24

**Authors:** Byung-Gun Kim, Chunwoo Yang, Kyungjoo Lee, Won Jun Choi

**Affiliations:** Department of Anesthesiology and Pain Medicine, School of Medicine, Inha University, Incheon, Republic of Korea.

**Keywords:** brachial plexus block, phrenic nerve palsy, spinal cord injury

## Abstract

**Rationale:**

Bilateral brachial plexus block (BPB) generally requires a relatively large dose of local anesthetic for a successful block, resulting in a high risk of local anesthetic systemic toxicity. It can also result in inadvertent bilateral phrenic nerve palsy, leading to respiratory failure. Hence, it has not been widely used. However, it can be performed in selected patients. In this report, we present a case of ultrasound-guided BPB for bilateral upper extremity surgery in a patient with cervical spinal cord injury (SCI).

**Patient concerns:**

A 25-year-old woman with SCI secondary to traumatic fifth cervical spine fracture scheduled for surgical treatment of bilateral elbow fracture received bilateral BPB.

**Diagnoses:**

Due to the complications of SCI, the patient had incomplete sensory loss, loss of motor function, and complete diaphragmatic paralysis on the right side.

**Interventions:**

Right infraclavicular and left axillary BPB was performed as the sole anesthetic procedure for bilateral upper extremity surgery.

**Outcomes:**

Bilateral BPB was successful for bilateral upper extremity surgery. The surgery was uneventful and without further complications.

**Lessons:**

Patients with cervical SCI have a high risk of respiratory complications. Bilateral BPB can be a suitable option for bilateral upper extremity surgery in selected patients. It is imperative to select an appropriate anesthetic technique that preserves respiratory function to minimize the potential risk of respiratory complications.

## 1 Introduction

Anesthetic management of a patient with cervical spinal cord injury (SCI) presents a great challenge to anesthesiologists. Acute cervical SCI is associated with several physiological disturbances including neurologic, cardiovascular, and respiratory systems,^[1]^ which might make the patient more susceptible to complications related to general anesthesia. Thus, it is important to avoid or reduce these risks in patients with SCI.

Regional anesthesia might be a better alternative to general anesthesia, especially for upper extremity surgery. However, bilateral brachial plexus block (BPB) can be challenging due to potential risk of local anesthetic systemic toxicity (LAST) and bilateral phrenic nerve palsy (PNP).^[2]^

In this case report, we present a case of ultrasound (US)-guided bilateral BPB for bilateral upper extremity surgery and discuss the potential problems and complications associated with bilateral BPB in a patient with cervical SCI.

## 2 Case presentation

Consent for publication was obtained from the patient. A 25-year-old woman (height 152 cm, weight 49 kg, American Society of Anesthesiologists physical status I) was admitted to our hospital for surgery of traumatic fifth cervical spine fracture with SCI. The operation was uneventful and without any complications. After surgery, she was transferred to the intensive care unit with continued sedation and intubation. On the fifth postoperative day, her trachea was extubated without dyspnea.

Ten days later, the patient was referred for surgical treatment of bilateral elbow fracture. Her medical history was unremarkable. No abnormalities were seen in the laboratory tests. However, chest x-ray showed an elevated right diaphragm and consolidations of right lower lung field that were not present on the chest radiograph at admission. On arterial blood gas analysis, pH was 7.427, partial pressure of oxygen was 65.6 mm Hg, and partial pressure of carbon dioxide was 33.5 mm Hg. Unfortunately, no pulmonary function tests were performed due to her condition. She had an impaired ability to cough and to clear the airway secretions effectively. Despite these findings, she complained of no dyspnea at that time. Preoperative neurological examination was normal for the left upper extremity. Although motor function was nearly absent in the radial, musculocutaneous, median, and ulnar nerve distributions of the right upper extremity, the sensory function was nearly intact in all nerve distributions. After a thorough discussion regarding the potential risks and benefits and approval of the patient, we decided to provide anesthesia with US-guided bilateral axillary BPB with neurostimulation, which avoids airway manipulations and respiratory failure associated with general anesthesia.

Standard monitors including noninvasive blood pressure, pulse oximetry, and electrocardiogram were applied and supplemental oxygen was administered using nasal prongs throughout the procedure. No sedatives or premedication were administered to avoid any interference with her lung function. While pre-procedural US examination of the right diaphragm showed no movement with the sigh and sniff test, a normal left diaphragmatic movement was observed (deep inspiration and sniff test, 7.6 and 4.2 cm, respectively).

Bilateral BPB was performed with US (Aloka Prosound SSD-3500SV, Hitachi Medical Ltd., Tokyo, Japan) and a 5 to 10 MHz linear probe with a nerve stimulator (Stimuplex HNS 12, B. Braun, Melsungen, Germany). The procedure was performed first on the right side. Since movements of the right elbow were impaired due to pain, we decided to perform infraclavicular BPB instead of axillary BPB. The US probe was positioned in a parasagittal plane medial to the coracoid process just below the clavicle and was adjusted to provide a transverse view of the axillary artery. Using an in-plane technique, an insulated needle (UniPlex NanoLine, Pajunk, Geisingen, Germany) was advanced to the posterior of the axillary artery and 25 mL of 0.5% ropivacaine was slowly injected with frequent aspiration. Thirty minutes later, the operation was commenced.

Five hours later, left axillary BPB was performed to reduce the risk of LAST. Under US guidance, an insulated needle was advanced until its tip was positioned dorsal to the artery. Twenty milliliter of 0.5% ropivacaine was slowly injected at this location. Subsequently, the needle was advanced toward the musculocutaneous nerve and 5 mL of 0.5% ropivacaine was deposited around it. Twenty minutes later, a complete sensorimotor block of the left arm was confirmed. No symptoms or signs of LAST were noted.

Since the patient was unable to cough effectively and unable to clear the secretions adequately, frequent oropharyngeal suction was required during the procedures. Her vital signs were stable and no opioid supplementation was needed during the surgery. Surgery was carried out uneventfully on both the sides. The patient required supplemental analgesics for postoperative pain on the right and the left side after 642 and 874 minutes, respectively. The postoperative pain was satisfactorily treated with ketorolac and tramadol.

## 3 Discussion

Various pathophysiological changes can occur after cervical SCI. In addition to neurologic injury, acute cervical SCI is usually associated with cardiovascular complications including neurogenic shock, autonomic dysreflexia, and other arrhythmias.^[3]^ Although the degree of respiratory dysfunction is dependent on the level and completeness of the injury, cervical SCI also results in respiratory dysfunction, which may include impaired respiratory muscles, diaphragmatic paralysis, reduced lung and chest wall compliance, impaired cough and secretion clearance, and increased work of breathing.^[4]^ Respiratory dysfunction is a leading cause of mortality and morbidity after SCI.^[5]^ Thus, it is important to reduce these risks in patients with SCI.

BPB can be a good alternative to general anesthesia, especially for upper extremity surgery. However, it can affect the respiratory function negatively. BPB at the level of the clavicle has a higher risk of pneumothorax, although US guidance can avoid this adverse effect. More importantly, BPB can result in inadvertent PNP and ipsilateral hemidiaphragmatic paralysis due to its close proximity to the phrenic nerve.^[6]^ The diaphragm is the primary muscle of inspiration, accounting for about 75% of the total tidal volume of respiration. A previous study reported that hemidiaphragmatic paralysis due to interscalene block can result in approximately 30% reduction in pulmonary function.^[7]^ Although well tolerated by healthy patients, PNP might result in potentially catastrophic consequences in a patient with compromised respiratory function. Thus, bilateral BPB has not been widely used due to a potential risk of bilateral PNP.

The incidence of PNP following BPB is associated with several factors, mainly dependent on the approach to the brachial plexus. It is 21% to 100% for interscalene BPB,^[8,9]^ 28% to 67% for supraclavicular BPB,^[10,11]^ and 5% to 13% for infraclavicular BPB.^[12,13]^ The effect of axillary BPB on the incidence of PNP has not yet been studied. However, since its injection site is anatomically far away from the phrenic nerve, its impact will likely be nonexistent. Axillary BPB provides surgical anesthesia for procedures involving hand, forearm, and elbow. Thus, it was deemed to be an optimal anesthetic technique to preserve the respiratory function in this patient.

Another major concern associated with bilateral BPB is the potential risk of LAST. Several preventive strategies to avoid LAST were pursued in this case. US guidance reduces the minimum effective dose of local anesthetic for a successful block,^[14,15]^ minimizing the risk of toxicity. In this case, we used a total ropivacaine dose of 250 mg, which was within the recommended safe single dose limit for peripheral nerve block. Further reduction in the dose was possible, but it might have sacrificed block success or duration. We used ropivacaine in this case, since it has a greater margin of safety among the long-acting local anesthetics.^[16,17]^ In addition, since simultaneous bilateral block might increase the peak plasma concentration of local anesthetic due to coincident absorption from each block site, we left a time gap between each block. A previous study recommended 60 minutes of time gap between each block to avoid toxicity.^[2]^

In conclusion, we have described a case of successful use of US-guided bilateral BPB as the sole anesthetic for bilateral upper extremity surgery in a patient with cervical SCI, with no further adverse effects. US-guided bilateral BPB can be safely used as an alternative to general anesthesia in patients with compromised respiratory function for bilateral upper extremity surgery.

## Author contributions

**Conceptualization:** C Yang, BG Kim.

**Data curation:** WJ Choi.

**Writing – original draft:** BG Kim, K Lee, WJ Choi.

**Writing – review and editing:** C Yang.
